# Supplementary material for: Detection of Superior Rice Genotypes and Yield Stability under Different Nitrogen Levels Using AMMI Model and Stability Statistics
Source: Plants (Basel). 2022 Oct 19;11(20):2775. doi: 10.3390/plants11202775 (PMC9611647; doi:10.3390/plants11202775)
Supplement: Supplementary file 1 [file plants-11-02775-s001.zip › plants-1962354-supplementary.pdf]

Supplementary Table S1. Genotypes GY means (g plant^{-1}) of the two successive seasons under the different N environments under study:

Genotypes	0N	LN	MN	HN	Mean
**1**	16.6	19.2	21.8	19.0	19.2
**2**	21.3	23.5	25.5	28.0	24.6
**3**	21.8	22.6	23.0	28.4	24.0
**4**	23.0	27.7	29.7	33.5	28.5
**5**	16.0	20.3	23.5	31.4	22.8
**6**	26.0	26.2	27.5	31.4	27.8
**7**	12.8	15.6	17.4	21.8	16.9
**8**	23.9	26.2	26.9	30.5	26.9
**9**	12.9	15.3	19.9	22.8	17.7
**10**	21.8	25.0	28.2	34.6	27.4
**11**	14.4	18.8	21.5	26.6	20.3
**12**	16.6	20.7	22.8	29.0	22.3
**13**	22.4	25.8	29.4	34.1	27.9
**14**	17.7	20.5	22.0	22.4	20.7
**15**	21.3	23.3	25.4	33.9	26.0
**16**	17.9	21.1	22.5	28.8	22.6
**17**	14.3	20.5	26.0	28.6	22.4
**18**	20.9	21.6	24.1	30.1	24.2
**19**	14.3	18.6	23.5	24.5	20.2
**20**	14.3	15.7	23.3	28.2	20.4
**21**	24.3	27.3	28.8	34.6	28.8
**22**	20.1	21.1	26.9	30.1	24.6
**23**	19.0	21.3	24.8	31.4	24.1
**24**	17.6	20.1	23.0	23.7	21.1
**25**	17.7	19.2	23.5	27.5	22.0
**26**	23.0	23.9	26.0	24.1	24.3
**27**	23.3	25.2	26.5	31.2	26.6
**28**	17.9	18.8	19.8	25.2	20.4
**29**	15.5	18.8	24.3	26.5	21.3

**30**	19.8	25.6	30.5	35.6	27.9
**31**	12.6	13.7	14.1	16.2	14.2
**32**	22.4	23.0	28.8	31.2	26.4
**33**	25.4	29.4	31.4	36.7	30.7
**34**	13.2	18.6	23.5	25.0	20.1
**35**	20.1	24.8	28.8	32.9	26.7
**36**	21.8	23.7	28.4	35.8	27.4
**37**	19.4	21.1	25.5	32.1	24.5
**38**	13.9	17.9	22.2	29.2	20.8
**39**	21.0	27.0	29.1	33.5	27.7
**40**	7.7	8.1	9.4	10.2	8.9
**41**	12.4	16.2	21.1	23.5	18.3
**42**	20.4	23.3	24.1	28.4	24.1
**43**	14.9	16.6	19.6	21.8	18.2
**44**	17.1	19.8	24.1	32.0	23.3
**45**	23.9	25.0	31.1	30.5	27.6
**46**	19.8	23.5	24.8	25.8	23.5
**47**	21.1	22.8	25.1	31.1	25.0
**48**	22.0	23.3	27.9	36.6	27.5
**49**	25.8	26.0	26.5	33.3	27.9
**50**	13.4	15.4	17.9	14.1	15.2
**51**	26.5	30.5	31.6	30.9	29.9
**52**	20.3	26.0	28.8	35.0	27.5
**53**	22.8	27.3	33.3	34.6	29.5
**54**	21.5	27.5	28.2	31.6	27.2
**55**	24.7	27.0	28.4	36.9	29.3

*Different N-levels (Urea form) to generate the different environments of no N (0N), Low N (LN), medium N (MN) and High N (HN) (i.e., 0, 48, 96, 165 Kg N ha^{-1}, respectively).

Supplementary Table S2. RCBD ANOVA table for the tested 55 genotypes under the different 4 N levels grown under 3 replicated

ENV	MEAN	DFG	MSG	FCG	PFG	DFB	MSB	FCB	PFB	DFE	MSE	CV	h2	AS
0N	19.13842	54	54.63788	61.34212	3.12E-61	2	1.821399	2.04489	0.134375	108	0.890707	4.931299	0.983698	0.991816
LN	21.94194	54	58.3741	85.21338	1.24E-68	2	1.13801	1.661245	0.194718	108	0.685034	3.772082	0.988265	0.994115
MN	24.93612	54	58.1975	60.19994	8.21E-61	2	1.722737	1.782012	0.173212	108	0.966737	3.942986	0.983389	0.99166
HN	28.83818	54	98.46499	104.403	2.99E-73	2	2.361273	2.50367	0.086521	108	0.943125	3.36757	0.990422	0.995199

Supplementary Table S3: AMMI analysis of variance for GY (g plant^{-1}) for the tested 55 genotypes under 4 different N levels.

Source	Df	Sum Sq	Mean Sq	F value	Pr(>F)	Proportion	Accumulated
ENV	3	8551.44	2850.48	1618.805	1.8E-11		
REP(ENV)	8	14.08684	1.760855	2.020717	0.042663		
GEN	54	12683.17	234.8735	269.5355	5.6E-299		
GEN:ENV	162	1879.254	11.60033	13.31228	2.9E-101		
IPCA1	56	1335.804	23.85364	27.37	0	71.1	71.1
IPCA2	54	407.2169	7.54105	8.65	0	21.7	92.8
IPCA3	52	136.2331	2.61987	3.01	0	7.2	100
Residuals	432	376.4452	0.871401				
Total	821	25383.65	30.91796				

ENV: Environments

REP: Replicates

GEN: Genotypes

IPCA: Interaction principal components analysis

DF: Degree of freedom

Sq: Squares

Pr: Propability.

Supplementary Table S4. GY means and IPCAs for the genotypes and the environments under study

Code	GY	IPCA1	IPCA2	IPCA3
**G1**	19.158	-1.269	-0.543	-0.057
**G10**	27.392	0.511	0.143	0.096
**G11**	20.342	0.340	-0.099	0.305
**G12**	22.300	0.390	0.130	0.360
**G13**	27.942	0.320	-0.103	0.020
**G14**	20.633	-0.876	-0.124	0.166
**G15**	25.958	0.551	0.683	0.011
**G16**	22.583	0.181	0.366	0.312
**G17**	22.350	0.590	-1.002	0.208
**G18**	24.175	0.045	0.596	-0.267
**G19**	20.217	0.006	-0.750	-0.048
**G2**	24.550	-0.474	0.095	0.028
**G20**	20.342	0.832	-0.278	-0.875
**G21**	28.750	0.077	0.354	0.273
**G22**	24.542	0.179	-0.162	-0.687
**G23**	24.133	0.498	0.256	-0.123
**G24**	21.083	-0.610	-0.257	-0.083
**G25**	21.967	0.103	0.051	-0.399
**G26**	24.267	-1.381	-0.126	-0.281
**G27**	26.525	-0.250	0.456	0.098
**G28**	20.425	-0.302	0.699	-0.044
**G29**	21.258	0.189	-0.570	-0.302
**G3**	23.950	-0.426	0.784	0.049
**G30**	27.883	0.878	-0.607	0.250
**G31**	14.142	-0.955	0.447	0.034
**G32**	26.350	-0.019	-0.167	-0.775
**G33**	30.725	0.199	0.080	0.374
**G34**	20.058	0.200	-0.878	0.138
**G35**	26.625	0.418	-0.432	0.205
**G36**	27.408	0.825	0.254	-0.355
**G37**	24.533	0.603	0.247	-0.325
**G38**	20.808	0.914	-0.072	0.070
**G39**	27.658	0.277	-0.323	0.669
**G4**	28.483	0.007	-0.154	0.471
**G40**	8.852	-1.104	0.314	-0.197
**G41**	18.300	0.181	-0.561	-0.119
**G42**	24.017	-0.307	0.305	0.313
**G43**	18.233	-0.432	0.015	-0.203
**G44**	23.242	0.925	0.210	-0.136
**G45**	27.633	-0.424	-0.579	-0.794
**G46**	23.483	-0.704	-0.183	0.344

**G47**	25.050	0.123	0.465	-0.079
**G48**	27.442	0.965	0.460	-0.455
**G49**	27.883	-0.232	1.019	-0.058
**G5**	22.775	0.899	0.118	0.263
**G50**	15.200	-1.532	-0.539	-0.171
**G51**	29.867	-0.998	-0.368	0.431
**G52**	27.517	0.690	-0.238	0.562
**G53**	29.492	0.258	-0.901	-0.161
**G54**	27.208	-0.154	-0.237	0.865
**G55**	29.258	0.459	0.736	0.197
**G6**	27.783	-0.603	0.637	-0.202
**G7**	16.900	-0.133	0.201	0.151
**G8**	26.875	-0.518	0.360	0.248
**G9**	17.725	0.069	-0.226	-0.315
**0N**	19.138	-2.354	1.844	-1.148
**HN**	28.838	3.691	1.099	0.072
**LN**	21.942	-1.391	-0.307	2.093
**MN**	24.936	0.055	-2.636	-1.017

GY: grain yield

IPCA: Interaction principal component analysis.

Supplementary Table S5. Ranking of the genotypes based on the different stability statistical analysis

GEN	GY_R	CV_R	ACV_R	POLAR_R	Var_R	Shukla_R	Wi_g_R	Wi_f_R	Wi_u_R	Ecoval_R	Sij_R	R2_R	ASTAB_R	ASI_R	ASV_R	AVAMGE_R	DA_R	DZ_R	EV_R	FA_R	MASI_R
G1	48	7	7	7	5	53	49	53	37	53	53	53	53	53	53	54	53	49	49	53	53
G10	16	32	34	34	42	21	7	10	9	21	7	7	20	9	9	24	18	24	24	18	8
G11	44	47	48	48	36	10	43	40	46	10	1	4	26	13	13	26	26	30	30	26	13
G12	37	42	41	41	38	15	34	36	39	15	16	13	9	29	29	19	19	3	3	19	29
G13	7	27	27	27	35	7	2	3	5	7	12	1	52	43	43	51	47	55	55	47	43
G14	42	5	4	4	6	42	41	49	31	42	23	48	6	5	5	5	6	10	10	6	5
G15	22	36	37	37	45	36	23	30	18	36	49	39	24	8	8	12	13	40	40	13	10
G16	35	34	33	33	29	9	30	38	32	9	27	25	17	31	31	25	23	7	7	23	31
G17	36	52	52	52	49	46	44	23	51	46	47	35	21	37	37	30	29	13	13	29	37
G18	28	25	25	25	22	18	28	31	33	18	40	38	4	4	4	3	2	14	14	2	4
G19	46	43	40	40	31	26	45	44	47	26	44	42	54	54	54	53	54	50	50	54	54
G2	24	9	9	9	12	19	25	26	15	19	10	2	15	33	33	22	21	5	5	21	33
G20	45	55	55	55	52	47	51	32	45	47	42	28	13	16	16	14	12	11	11	12	16
G21	5	18	18	18	23	6	5	7	3	6	24	22	27	22	22	27	28	28	28	28	22
G22	25	31	31	31	32	13	24	19	35	13	33	33	22	15	15	17	20	26	26	20	15
G23	29	39	38	38	41	23	27	27	28	23	21	16	33	30	30	38	32	36	36	32	30
G24	40	15	15	15	11	29	35	46	34	29	25	41	47	45	45	47	48	43	43	48	45
G25	38	33	32	32	27	2	33	28	38	2	13	15	45	49	49	50	49	35	35	49	49
G26	27	1	1	1	2	54	37	50	21	54	36	54	29	2	2	16	16	46	46	16	2
G27	20	11	13	13	17	12	17	14	10	12	18	31	5	11	11	6	5	12	12	5	11
G28	43	22	22	22	15	28	40	47	40	28	38	45	37	20	20	28	31	38	38	31	20
G29	39	44	44	44	34	20	39	34	41	20	35	30	19	27	27	21	24	18	18	24	27
G3	31	10	11	11	13	32	32	33	27	32	41	49	7	21	21	8	10	8	8	10	21
G30	8	46	47	47	55	48	16	2	29	48	17	6	40	42	42	41	41	33	33	41	42
G31	54	6	5	5	3	49	53	52	52	49	5	29	25	36	36	29	30	22	22	30	36
G32	21	23	24	24	24	16	15	17	26	16	39	36	38	47	47	44	44	23	23	44	47

G33	1	21	21	21	30	5	1	1	2	5	9	12	28	17	17	20	22	44	44	22	17
G34	47	49	49	49	39	31	46	41	53	31	46	40	11	1	1	4	4	21	21	4	1
G35	19	35	35	35	44	24	10	6	24	24	14	9	50	52	52	52	52	39	39	52	52
G36	15	40	42	42	48	41	11	13	17	41	37	21	18	14	14	13	17	19	19	17	14
G37	26	41	43	43	46	30	29	22	30	30	30	19	14	18	18	18	11	15	15	11	19
G38	41	54	54	54	53	44	47	42	48	44	2	3	8	26	26	10	14	4	4	14	26
G39	11	30	30	30	37	22	8	4	19	22	34	26	43	48	48	46	45	29	29	45	48
G4	6	19	20	20	25	4	4	5	6	4	19	20	16	25	25	15	15	17	17	15	25
G40	55	14	10	10	1	52	55	54	55	52	8	18	46	28	28	40	37	53	53	37	28
G41	49	50	50	50	33	17	52	43	54	17	31	24	30	41	41	36	35	27	27	35	41
G42	30	17	17	17	16	11	26	24	14	11	15	23	10	10	10	11	8	9	9	8	7
G43	50	24	23	23	14	14	42	48	43	14	4	8	49	50	50	48	50	48	48	50	50
G44	33	51	51	51	51	45	36	35	36	45	32	17	44	24	24	35	40	47	47	40	24
G45	12	16	16	16	19	37	14	29	13	37	52	50	41	46	46	43	43	31	31	43	46
G46	32	8	8	8	9	35	31	39	20	35	28	47	55	55	55	55	55	54	54	55	55
G47	23	26	26	26	26	8	21	21	23	8	29	32	48	51	51	49	51	45	45	51	51
G48	14	45	46	46	54	50	18	18	22	50	50	34	39	40	40	33	39	41	41	39	40
G49	9	12	14	14	18	40	20	20	12	40	54	51	42	23	23	31	34	42	42	34	23
G5	34	53	53	53	50	43	38	37	42	43	22	10	2	19	19	7	7	1	1	7	18
G50	53	13	12	12	4	55	54	55	44	55	55	55	36	7	7	23	25	52	52	25	9
G51	2	2	2	2	7	51	13	25	1	51	45	52	32	32	32	32	33	37	37	33	32
G52	13	38	39	39	47	39	9	8	25	39	20	11	34	38	38	39	38	34	34	38	38
G53	3	29	29	29	43	34	3	9	8	34	48	37	1	6	6	2	1	2	2	1	6
G54	17	20	19	19	21	25	12	12	16	25	43	44	23	34	34	34	27	20	20	27	34
G55	4	28	28	28	40	33	6	16	4	33	51	43	3	3	3	1	3	6	6	3	3
G6	10	3	3	3	8	38	22	11	11	38	26	46	35	44	44	42	42	25	25	42	44
G7	52	37	36	36	20	1	48	51	50	1	3	5	31	35	35	37	36	32	32	36	35
G8	18	4	6	6	10	27	19	15	7	27	6	27	12	12	12	9	9	16	16	9	12
G9	51	48	45	45	28	3	50	45	49	3	11	14	51	39	39	45	46	51	51	46	39

Continue Supplementary Table S5

GEN	MASV_R	SIPC_R	ZA_R	WAAS_R	WAASB_R	HMGV_R	RPGV_R	HMRPGV_R	Pi_a_R	Pi_f_R	Pi_u_R	Gai_R	S1_R	S2_R	S3_R	S6_R	N1_R	N2_R	N3_R	N4_R
G1	53	52	54	53	54	46	48	48	48	51	41	48	52.5	52	45	53	30	14	21	41
G10	23	19	10	8	8	27	28	28	14	12	15	13	28.5	10	10	7	4.5	28	35	45
G11	29	14	8	7	7	45	45	45	43	43	44	44	15	8	15	27	6	4	4	12
G12	18	4	22	24	24	24	24	24	36	33	36	36	38	11	27	38	17	8	7	29
G13	43	54	47	47	47	47	46	46	7	4	12	9	2	1	5	4	7	34	33	4
G14	9	10	6	6	6	5	5	5	42	48	34	41	38	35	46	52	41	19	18	25
G15	7	23	12	11	11	26	26	25	22	20	24	22	54	37	29	20	44	38	38	47
G16	22	17	27	30	30	31	31	31	35	36	33	34	44	13	26	32	14	12	8	35
G17	28	21	32	34	34	39	39	39	37	28	42	38	20.5	47	52	41	47	30	30	18
G18	1	3	2	3	3	37	38	37	27	30	29	28	38	25	12	10	37.5	29	24	37
G19	54	48	53	54	53	25	25	27	45	44	45	45	48.5	28	32	40	30	10	10	34
G2	20	12	25	28	28	15	14	13	25	31	23	24	17.5	16	39	28	23	31	23	20
G20	19	13	17	17	18	19	19	19	46	34	49	46	1	55	47	54	55	32	26	1
G21	30	24	26	25	25	42	42	42	5	9	3	5	26	6	4	3	1.5	42	45	49
G22	24	27	19	16	16	41	40	40	24	24	30	26	15	22	20	18	17	18	27	19
G23	37	35	33	32	32	28	29	30	28	26	32	31	34	15	16.5	19	14	16	20	33
G24	48	47	49	48	48	13	11	12	40	45	35	39	44	19	19	31	11	7	9	31
G25	47	43	48	49	49	54	54	54	38	38	38	37	11.5	9	7	11	4.5	5	6	7
G26	6	22	4	2	2	20	21	21	31	41	16	25	48.5	50	55	48	43	39	37	44
G27	5	8	7	9	10	1	1	1	20	21	13	19	15	26	18	16	36	40	39	27
G28	35	31	24	23	23	48	47	47	44	46	39	42	23	29	49	46	26.5	13	12	14
G29	25	25	28	29	29	21	20	20	39	39	43	40	31	24	36	39	24	11	13	23
G3	10	11	16	18	17	43	44	44	30	35	25	30	23	43	51	42	50	33	32	22
G30	40	42	43	43	44	17	17	16	9	3	18	12	6	46	48	34	51.5	52	50	9
G31	27	30	34	36	36	30	27	26	54	54	54	54	6	53	3	35.5	54	23	15	2
G32	44	26	41	44	43	44	43	43	21	16	20	21	34	21	30	24	26.5	35	36	43

G33	17	36	23	20	20	11	12	11	1	1	2	1	23	3	2	2	9	54	55	54
G34	2	6	1	1	1	6	6	6	47	42	47	47	41	34	43	49	39	15	14	26
G35	52	46	52	52	52	55	55	55	19	13	22	20	8.5	18	28	17	20.5	37	34	10
G36	21	16	15	15	15	50	50	50	16	8	19	16	36	39	22	13	40	44	47	46
G37	14	20	20	19	19	29	30	29	26	23	31	27	28.5	23	34	26	19	21	29	32
G38	15	7	18	21	22	49	49	49	41	37	46	43	25	48	54	55	51.5	24	22	15
G39	45	37	45	46	46	33	33	33	8	11	14	11	3.5	20	24	14	17	45	42	6
G4	13	18	21	22	21	36	36	36	6	7	6	6	8.5	4	9	6	3	41	43	21
G40	31	49	36	33	33	10	10	10	55	55	55	55	17.5	51	1	1	53	17	11	11
G41	32	32	38	38	38	32	32	32	49	47	51	50	28.5	14	31	51	12	6	5	16
G42	16	9	9	10	9	23	23	23	29	32	27	29	3.5	17	21	25	28	26	19	3
G43	49	53	51	50	51	18	16	17	50	50	48	49	28.5	12	35	50	10	3	3	17
G44	46	39	30	26	26	8	7	7	33	27	37	33	44	45	53	43	45	27	31	39
G45	42	38	44	45	45	35	35	35	13	15	11	10	51	40	42	33	48.5	50	46	51
G46	55	55	55	55	55	53	53	53	32	40	28	32	32	31	41	37	25	25	25	30
G47	50	50	50	51	50	2	2	2	23	25	26	23	34	7	6	8	14	22	16	38
G48	33	45	39	40	40	16	13	14	17	10	21	17	50	49	38	21	42	43	48	50
G49	39	40	29	27	27	3	3	3	10	18	7	7	40	36	25	12	35	46	49	48
G5	8	1	11	14	14	9	9	8	34	29	40	35	46	41	50	45	33	20	28	40
G50	11	34	14	12	12	12	15	15	53	53	50	53	55	54	16.5	47	37.5	9	17	36
G51	34	41	35	35	35	4	4	4	3	14	1	2	47	44	44	29	33	55	53	53
G52	38	44	40	39	39	7	8	9	11	5	17	14	19	30	37	22	20.5	48	44	42
G53	3	2	5	5	5	52	52	52	2	2	8	3	42	38	11	5	30	51	54	52
G54	26	28	31	31	31	14	18	18	15	17	10	15	13	27	23	15	22	36	41	28
G55	4	5	3	4	4	51	51	51	4	6	4	4	52.5	32	13	9	33	53	52	55
G6	41	29	42	42	42	40	41	41	12	19	5	8	11.5	42	33	23	46	49	51	24
G7	36	33	37	37	37	22	22	22	52	52	52	52	10	2	8	35.5	8	2	1	5
G8	12	15	13	13	13	34	34	34	18	22	9	18	6	33	40	30	48.5	47	40	8
G9	51	51	46	41	41	38	37	38	51	49	53	51	20.5	5	14	44	1.5	1	2	13

Supplementary Table S6 Physical and chemical properties of the experimental soil before rice transplanting at the two growing seasons:

**Soil properties**	**Values**	
**Mechanical:**		
**Clay (%)**	55.8	56.0
**Silt (%)**	32.**0**	32.0
**Sand (%)**	12.2	12.0
**Texture**	Clayey	Clayey
**Chemical:**		
**Total nitrogen (mg kg^{-1})**	759	770
**Available P (mg kg^{-1}) (0.5 M NaHCO3)**	27.14	27.0
**Available Potassium (ppm)**	340	400
**pH (1: 2.5 soil suspension)**	8.19	8.30
**EC dS/m (soil extraction)**	0.66	1.01
**Available Fe**	5.8	5.9
**Available Zn**	1.05	1.04

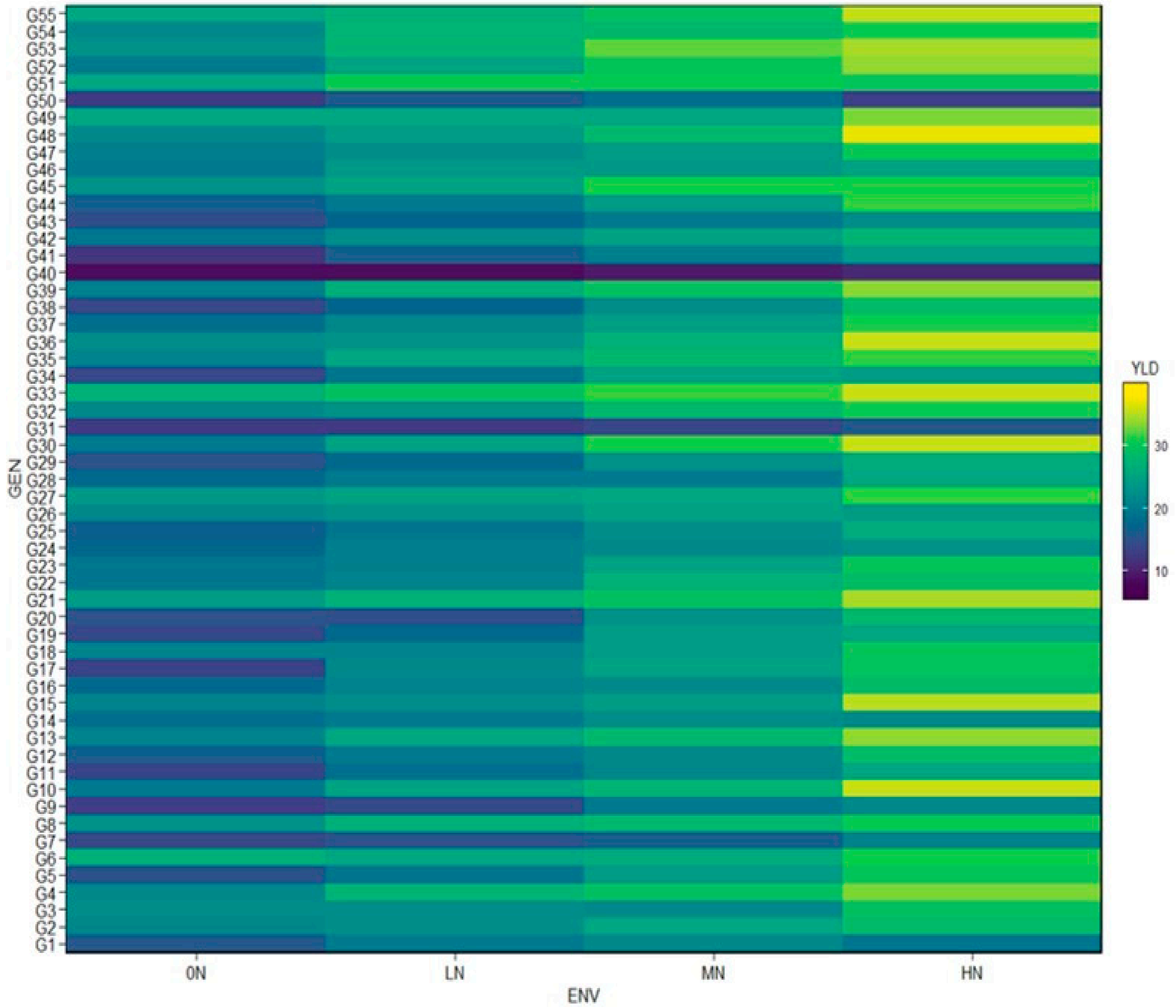

Supplementary Figure S1: heat map of the GY performance of the tested 55 genotypes across the 4 different environments, genotypes and environments legends are presented in Table 2.
